# Canonical Decomposition of Ictal Scalp EEG and Accurate Source Localisation: Principles and Simulation Study

**DOI:** 10.1155/2007/58253

**Published:** 2007-11-27

**Authors:** Maarten De Vos, Lieven De Lathauwer, Bart Vanrumste, Sabine Van Huffel, W. Van Paesschen

**Affiliations:** ^1^ESAT-SISTA, Katholieke Universiteit Leuven, Kasteelpark Arenberg 10, 3001 Heverlee-Leuven, Belgium; ^2^CNRS-ETIS, 6 Avenue du Ponceau BP 44, 95014 Cergy-Pontoise, France; ^3^Department of Neurology, University Hospital Gasthuisberg, Katholieke Universiteit Leuven Herestraat 49, 3000 Leuven, Belgium

## Abstract

Long-term electroencephalographic (EEG) recordings are important in the presurgical evaluation of refractory partial epilepsy for the delineation of the ictal onset zones. In this paper, we introduce a new concept for an automatic, fast, and objective localisation of the ictal onset zone in ictal EEG recordings. Canonical decomposition of ictal EEG decomposes the EEG in atoms. One or more atoms are related to the seizure activity. A single dipole was then fitted to model the potential distribution of each epileptic atom. In this study, we performed a simulation study in order to estimate the dipole localisation error. Ictal dipole localisation was very accurate, even at low signal-to-noise ratios, was not affected by seizure activity frequency or frequency changes, and was minimally affected by the waveform and depth of the ictal onset zone location. Ictal dipole localisation error using 21 electrodes was around 10.0 mm and improved more than tenfold in the range of 0.5–1.0 mm using 148 channels. In conclusion, our simulation study of canonical decomposition of ictal scalp EEG allowed a robust and accurate localisation of the ictal onset zone.

## 1. INTRODUCTION

Epilepsy is one of the most common, severe neurological diseases. People suffering from epilepsy, who are not helped by medication, can potentially benefit from epilepsy surgery [1]. In order to remove the epileptogenic region, a precise localisation of the epileptic focus is mandatory. One of the diagnostic tools to localize this region of seizure onset zone is recording of ictal scalp electroencephalogram (EEG) [2]. The EEG measures electric potential distributions at discrete recording sites on the scalp. These potential distributions are the direct consequence of internal electrical currents associated with the synchronous firing of neurons. EEG recordings have an excellent temporal resolution, but a rather poor spatial accuracy due to the limited number of recording sites and the shielding effect of the skull. Visual analysis of EEG recordings aims to determine which lobe or which electrodes are activated. A challenging problem in neuroscience is to estimate in a more objective and precise way the regions of the brain that are active, given only the measured potential distributions.

Estimating the electrical source in the brain from the scalp EEG is a difficult problem since an infinite number of internal electrical currents can generate the same potential distribution on the scalp. Several different approaches to solve this source localisation or inverse problem exist based on different assumptions [3, 4]. One assumption is that the surface potentials are generated by a dense set of dipolar sources distributed on the cortical surface. The most popular method from this “distributed source” family is Loreta [5]. In a second approach, which is the most common, a limited number of “equivalent dipoles” are assumed to generate the measured potential distribution [6]. Dipole modeling is a well-established technique for localising interictal spikes, see, for example, [7, 8] and references herein. Ictal EEG recordings have been subjected to dipole modeling much less often than interictal spikes. The seizure discharge is a very complex pattern. Mainly artifacts, such as electromyogram, movement, eye blinks, and eye movements artifacts, render modeling difficult [9]. Even visual analysis of seizure onset can be significantly improved by removing muscle artifacts [10]. Moreover, the low signal-to-noise ratio of the seizure signal can render the correct localisation very diffuse. However, when source localisation of seizure onset would be possible, it can reduce the need for invasive intracranial EEG recordings. So far, the results of ictal EEG source localisation have been discouraging. One study reports that the used “inverse solution” [11] is not useful at all for localising seizure onsets [12]. Some studies were restricted to temporal lobe seizures [13, 14]. One reason to select temporal lobe seizures is that source analysis is most reliable during periods of relative signal stationarity in order to average repetitive ictal waveforms, which is more common in temporal than in extratemporal lobe seizures. Another reason for selecting only temporal lobe seizures is that extratemporal lobe seizures are much more frequently contaminated by severe artifacts. Two other studies were not restricted to temporal lobe seizures. Gotman [9] obtained reliable models for seizure onset in 6 out of 15 patients (40%) and Boon et al. [15] in 31 out of 100 patients (31%). In the latter study, the ictal EEG was filtered with a narrowband filter (1–14 Hz), while ictal seizure activity is known to consist of rhythmical waves with a frequency between 3 and 29 Hz [16]. Filtering should be avoided because these filters suppress all high-frequency activity, including electrical brain activity. Moreover, muscle artifacts filtered by a lowpass filter can resemble cerebral activity [17]. All these studies illustrate how difficult it is to reliably estimate ictal sources, and indicate that the current ictal scalp EEG source analysis tools can not be used for a reliable localisation of the ictal onset zone during presurgical evaluation. A recent study on source analysis developed a novel integrative approach to characterise the structure of seizures in the space, time, and frequency domains and showed some promising results [18].

The localising value of dipole modeling of ictal EEG can be improved by first removing artifacts and afterwards estimating the sources [19]. Another possibility is to decompose the measured EEG in a sum of individual contributions of distinct brain sources and localising the epilepsy-related source in order to estimate the epileptic focus. Space-time decomposition techniques like principal component analysis (PCA) and independent component analysis (ICA) of multichannel EEG can be used for artifact removal [20, 21] or for extracting activities of interest [22, 23]. However, in order to obtain a matrix decomposition like PCA and ICA, assumptions like orthogonality or independence—which are physically maybe irrelevant—have to be imposed. Recently, we have shown that a space-time-frequency decomposition of a three-way array containing wavelet-transformed EEG by the canonical decomposition (Candecomp), also known as parallel factor analysis (Parafac), reliably separated a seizure atom from the noise and background activity with a sensitivity of more than 90% [24]. This work was inspired by [25, 26]. The main advantage of this decomposition is that no extra assumptions have to be imposed. After the decomposition, the potential distribution over the electrodes of the epilectical activity was obtained, and displayed as a 2D image. Electrodes with large potential amplitudes could be considered as close to the focus. The aim of the present study was twofold. First, we wanted to investigate whether it was possible to localise the ictal onset zone in the head by applying dipole source localisation after canonical decomposition of ictal EEG recordings. Second, we wanted to investigate the accuracy of this localising method with realistic simulations under different conditions. We were especially interested (i) in the influence of the frequency of the seizure activity on the localisation, (ii) how the dipole localisation would be influenced by changes in frequency, and (iii) if the dipole estimation accuracy could be improved by increasing the number of electrodes.

We start by revising the canonical decomposition of a higher-order array (Section 2.1). We then define how we constructed realistically simulated EEG (Section 2.2), assessed the accuracy of our method (Section 3) and finally discuss our results (Section 4).

## 2. MATERIALS AND METHODS

### 2.1. Method

In our application, a three-way data array X with dimensions (space, scale, time) is obtained by wavelet-transforming every channel of the original (or simulated) EEG matrix. The continuous wavelet transform C at scale a and time t of a signal x(t) is defined as

(1) $$C(a,t)=\int_{-\infty }^{\infty }x(t)\phi^{⋆}(a,t,\tau )d\tau$$

with $\phi^{⋆}$ the chosen wavelet. Different real wavelets can be used. In this study, we used a biorthogonal wavelet with decomposition order 3. From the scale a of the wavelet, the frequency f of the signal can be estimated as

(2) $$f\approx \frac{{\;f}_{c}}{\left(a\Delta t\right)}$$

with $f_{c}$ the center frequency of the wavelet and Δt the sampling period.

**Figure 1:**
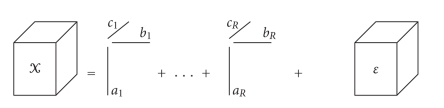
The Candecomp model with R components.

The trilinear Candecomp [27–29] is a generalisation of the singular value decomposition (SVD) for higher orders. It is defined for a three-way array X(I×J×K) as

(3) $$x_{ijk}=\overset{R}{\underset{r=1}{\sum }}a_{ir}b_{jr}c_{kr}+e_{ijk},$$

where R is the number of components used in the Candecomp model and $e_{ijk}$ are the residuals containing the unexplained variation. A pictorial representation of the Candecomp model is given in Figure 1. The Candecomp model is a trilinear model: fixing the parameters in two modes, $x_{ijk}$ is expressed as a linear function of the remaining parameters. Another equivalent and useful expression of the same Candecomp model is given with the Khatri-Rao product ⊙, defined as the column-wise Kronecker product [30].

Stack the elements of the tensor $X^{I\times J\times K}$ in a matrix $X^{IJ\times K}$ as

(4) $$X_{(i-1)J+j,k}=x_{ijk}.$$

Construct a matrix E in a similar way. Collect the elements $a_{ir}$ in A; $b_{jr}$ in B and $c_{kr}$ in C. Then

(5) $$X^{IJ\times K}=(A^{I\times R}⊙B^{J\times R})(C^{K\times R}{)}^{T}+E^{IJ\times K}.$$

Comparing the number of free parameters of a generic tensor and a Candecomp model, it can be seen that this model is very restricted. The advantage of this model is its uniqueness under mild conditions [31–33]:

(6) $$k_{A}+k_{B}+k_{C}⩾2R+2$$

with $k_{M}$ the k-rank of matrix M. The k-rank of matrix M is defined as the maximal number r such that any set of r columns of M is linearly independent. For tensors of which one dimension is greater than the rank, another less restrictive condition has recently been derived in [34].

The canonical decomposition is usually computed by means of an alternating least-squares (ALS) algorithm [30]. This means that the least-squares cost function

(7) $$f(A,B,C)={\left\|X-\overset{R}{\underset{r=1}{\sum }}A_{r}\circ B_{r}\circ C_{r}\right\|}^{2}$$

is minimized by means of alternating updates of one of its matrix arguments, keeping the other two matrices fixed. Because the canonical decomposition is a multilinear decomposition, each update just amounts to solving a classical linear least-squares problem. The convergence may be local. To increase the probability that the global minimum is found, the algorithm is reinitialized a couple of times. Since the introduction of the ALS algorithm, other computational schemes have been proposed [34–37].

**Figure 2:**
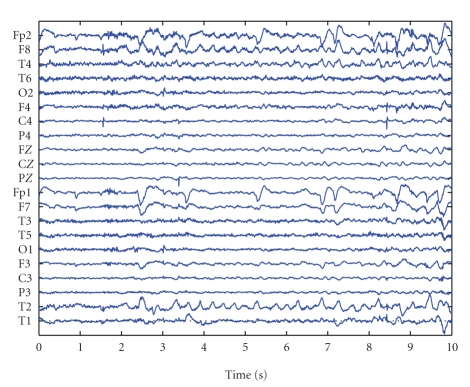
10 seconds of EEG containing the start of a seizure.

**Figure 3:**
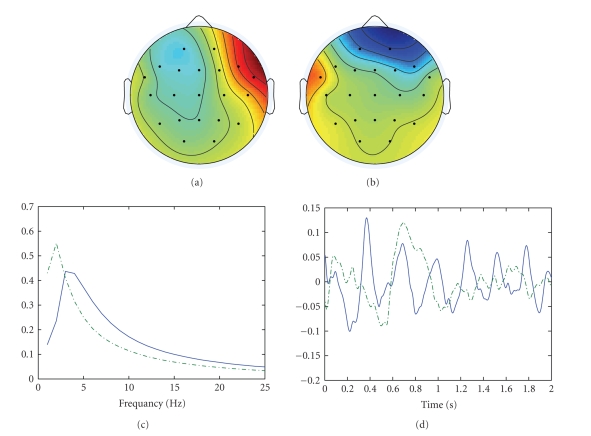
Seconds 3 to 5 of the seizure shown in Figure 2 are decomposed with the canonical decomposition with 2 atoms. (a) (b) the spatial potential distributions of the two atoms. (c) The frequency content of the atoms. (d) The time course of the atoms. First atom drawn in solid line correspond with (a). Dash-dotted line correspond with (b). First atom is seizure atom.

**Figure 4:**
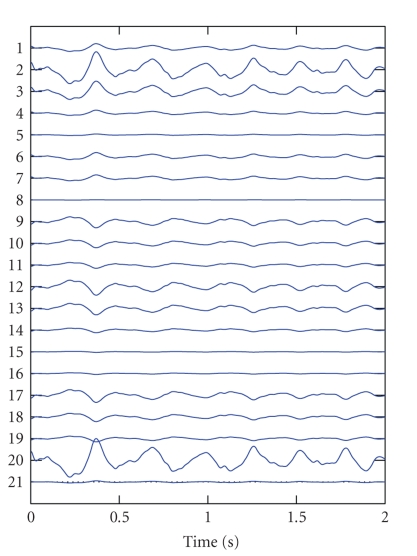
The seizure atom from Figure 3 is reconstructed in EEG coordinates after canonical decomposition.

When Candecomp is used for seizure localisation, 2 seconds of EEG at the seizure onset is wavelet transformed. The obtained three-way array is decomposed with Candecomp with R atoms. Several techniques exist to determine the optimal number of atoms [30]. Corcondia was used to determine the optimal number of atoms R. After decomposition, each atom has a component in the space ($a_{i}$), time ($b_{i}$), and frequency domain ($c_{i}$). The seizure atom(s) can be selected based on characteristic signatures in the different domains. At the ictal onset, seizure activity is recognised by rhythmical activity that is well localised in space and frequency. This was also described in [38]. Another possibility is to reconstruct the decomposed atoms in EEG settings by means of the inverse continuous wavelet transform (ICWT) [39]. We illustrate this approach with an example. EEG containing clear ictal activity in the right temporal lobe is given in Figure 2. The seizure starts at Second 3, and the EEG between Second 3 and 5 is wavelet transformed and decomposed with Candecomp (see Figure 3). Corcondia indicated that a decomposition in two atoms would be appropriate. The first atom is recognized as seizure atom. The frequency component peaks around 3 Hz and the time component is a rhythmical waveform that increases in amplitude. When this component is reconstructed in EEG settings, pure ictal activity can be seen (see Figure 4). Because the atoms in the canonical decomposition have a very simple, trilinear structure, we propose to fit only 1 dipole for every atom. We expect that, when a patient suffers from multifocal epilepsy, different atoms will be related to activity generated by the different dipoles.

Dipole estimation then determines the dipole's coordinates and orientation that best generate the given potential distribution in a least-squares sense. For computational simplicity, we used a spherical head model in this study.

### 2.2. Simulation

Consider a matrix X of dimension 500-by-21 representing a 21-channel EEG section of 2.0 seconds long. Each vector $x_{s},\;s=1,\ldots ,21$ of X contains the time course of an EEG channel:

(8) $$X={\left[x_{1},x_{2},\ldots ,x_{21}\right]}^{T}.$$

In this simulation study X includes both seizure activity, and superimposed noise. Both signals are described as follows.

#### 2.2.1. Synthetic seizure activity

The EEG of the ictal activity was generated using a fixed dipole in a three-shell spherical head model. The different time courses generated by the dipole are described below. The amplification factors at each electrode were computed by solving the forward problem for a dipole in a three-shell spherical head model consisting of a brain, a skull, and a scalp compartment [40]. Each compartment had a specific conductivity with a ratio equal to 1:1/16:1 for the brain, skull, and scalp compartment, respectively [41]. The brain and scalp conductivity was $3.3\times 10^{-4}/\Omega mm$ [42]. Radii of the outer boundary of the brain, skull, and scalp region equal to, respectively, 8 cm, 8.5 cm and 9.2 cm were used. Anumber of 21 electrodes were used: *Fp2, F8, T4, T6, O2, F4, C4, P4, Fz, Cz, Pz, Fp1, F7, T3, T5, O1, F3, C3*, and *P3* placed according to the 10–20 system for electrode placement [43] and additional electrodes T1 and T2 on the temporal region. The time course of the scalp potentials was stored in a 500-by-21 dimensional matrix A, representing 2 seconds of EEG with sample frequency of 250 Hz.

Unless otherwise stated, dipole coordinates x (left ear to right ear), y (posterior to anterior) and z (up, through the Cz electrode) were [−0.5 0 0.1] and the dipole orientations $d_{x},\;d_{y}$, and $d_{z}$ were [1 0 0].

**Figure 5:**
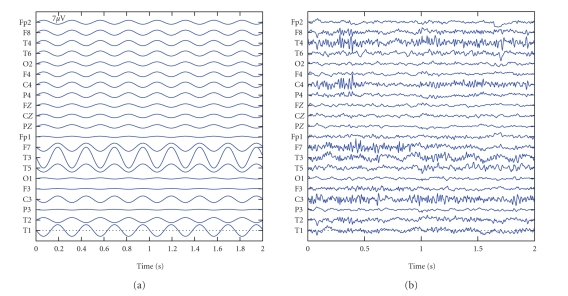
Simulated data. (a) The time course of the scalp potentials reflecting the 4 Hz epileptiform activity on each electrode. (b) The simulated data matrix for an SNR equal to 0.7.

The following seizure characteristics were simulated:

- Seizure activity in patients with mesial temporal lobe epilepsy (MTLE) is typically expressed by a 4 Hz sinusoidal waveform [44]. In a first simulation we estimated the dipole localisation error when seizure activity was represented by a 4 Hz sinusoid at different noise levels (see Figure 5(a)). We also investigated the influence of the specific waveform and estimated the localisation error when seizure activity was represented by a 4 Hz sawtooth, instead of a sinusoidal wave, at different noise levels.
- Ictal EEG activity can have a frequency in the delta, theta, alpha, or beta range. In a second simulation, therefore, we estimated the influence of the frequency of the seizure signal on ictal scalp EEG source localisation at a fixed noise level. We were particularly interested if the possible overlap in frequency content between faster ictal activity and seizure activity would bias the decomposition and thus the dipole estimate.
- Epileptic seizure activity can rapidly change in frequency. Ictal EEG activity is often characterized by low-voltage fast activity in the beta range which gradually slows down to alpha or theta frequencies with increasing amplitude. The canonical decomposition exploits frequency information during the decomposition. In order to test possible shortcomings of the canonical decomposition of ictal EEG, we wanted to estimate the accuracy when the model is violated. In a third simulation, we assessed the dipole localisation error when the frequency changed during the 2 seconds under investigation. This does not give a trilinear signal after wavelet transformation. We simulated a chirp that linearly changed in frequency from 8 Hz at the start to 4 Hz at the end of the considered 2 seconds. The signal also doubled in amplitude.
- In our previous study [24], two atoms were obtained after the decomposition of in vivo seizures and a distinction could be made between a seizure and a nonseizure atom. An interesting question is how well different dipoles generating similar ictal signals will be distinguished from each other. Such activity can be measured in the case of multifocal epilepsy. In a fourth simulation, we considered two rhythmical sources firing at the same frequency separated from each other by about 1 cm: the second dipole had coordinates [−0.4 0 0.1]. These dipoles generated similar potential distributions at the scalp.
- In a fifth simulation, the influence of the dipole localisation was investigated. Deeper sources generate a weaker signal captured by the electrodes and are possibly less accurately separated from background EEG. We varied the z-coordinate of the dipole between 0 and 0.8. x and y were kept fixed at −0.5 and 0, respectively.
- 21-channel EEG does not have an optimal spatial resolution due to the low spatial sampling. In a last simulation, we investigated how much the dipole localisation error could be improved by using dense array EEG [45]. We used 148 electrodes, uniformly distributed over the realistic domain of the same spherical head model.

#### 2.2.2. Noise

A 500-by-21 noise matrix B contained 2 seconds of awake background EEG activity, recorded with the same electrode configuration as in (A), from a normal subject. On this matrix B, muscle artifacts were superimposed. These muscle artifacts were separated from contaminated background activity using BSS-CCA [46]. For the last simulation with dense-array EEG, the noise was Gaussian, because no background EEG was available with this high number of electrodes.

#### 2.2.3. The simulated signal

In the simulation study the noise matrix B is superimposed on the signal matrix A containing the epileptical activity:

(9) $$\begin{array}{l} X(\lambda )=A+\lambda \cdot B \end{array}$$

with λ∈ℝ. The root mean-squared (RMS) value of the signal is then equal to

(10) $$\begin{array}{l} \text{RMS}(A)=\sqrt{\frac{1}{S\cdot N}\overset{S}{\underset{s=1}{\sum }}\;\overset{N-1}{\underset{n=0}{\sum }}{\left(A(n,s)\right)}^{2}} \end{array}$$

with N the number of time samples; and the RMS value of the noise is equal to

(11) $$\begin{array}{l} \text{RMS}(\lambda \cdot B)=\sqrt{\frac{1}{S\cdot N}\overset{S}{\underset{s=1}{\sum }}\overset{N-1}{\underset{n=0}{\sum }}{\left(\lambda \cdot B(n,s)\right)}^{2}}. \end{array}$$

The signal-to-noise ratio (SNR) is then defined as follows:

(12) $$\begin{array}{l} \text{SNR}=\frac{\text{RMS}(A)}{\text{RMS}(\lambda \cdot B)}. \end{array}$$

Changing the parameter λ alters the noise level of our simulated signal.

## 3. RESULTS

**Figure 6:**
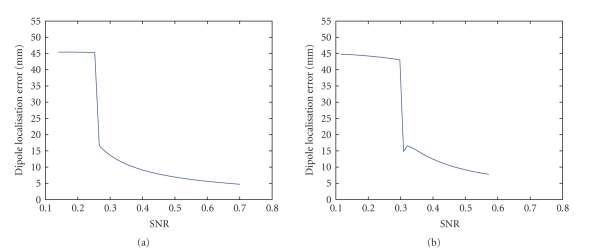
(a) The dipole localisation error in function of the noise level when a sinus waveform was used as epileptic signal. (b) Idem as (a) but a sharp wave was used as epileptic signal.

Figure 6(a) shows the dipole localisation error in function of the SNR when one dipole was fitted on the potential distribution extracted with Candecomp. At an SNR of 0.4, the localisation error became smaller than 1 cm and at an SNR of 0.7, the error between the simulated and the fitted dipole was only 5 mm. At SNRs lower than 0.26, there was no atom that clearly corresponded to the seizure activity as can be seen by the large localisation error. At higher noise levels, one atom contained pure rhythmical activity as can be seen by the sudden improvement in dipole localisation error. Figure 6(b) shows the dipole fit error when a sawtooth was used to simulate ictal EEG. The error was slightly larger compared to the perfect sinusoidal signal, but still in the same range.

**Figure 7:**
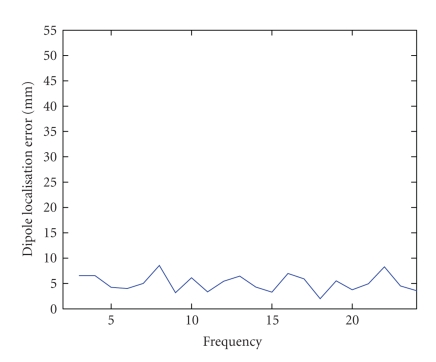
The dipole localisation error as a function of the seizure frequency.

Figure 7 shows the dipole localisation error for different frequencies of the simulated epileptic signal at an SNR of 0.7 (see Figure 5(b)). From this figure, it can be seen that the accuracy of the separation of ictal EEG and the dipole fit does not depend on the frequency of the signal. At all frequencies, a dipole is fitted with an error smaller than 1 cm. This means that even when the frequency content of ictal activity overlaps with frequency content of muscle artifacts, a good separation is obtained.

**Figure 8:**
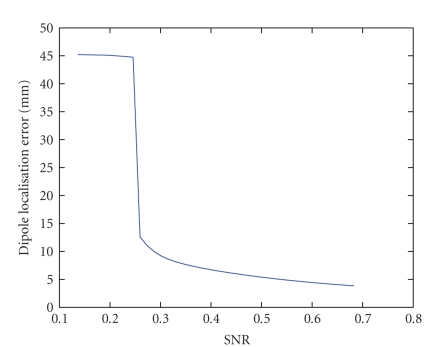
The dipole localisation error as a function of the noise level, when the seizure activity is changed in frequency during the time interval under investigation.

**Figure 9:**
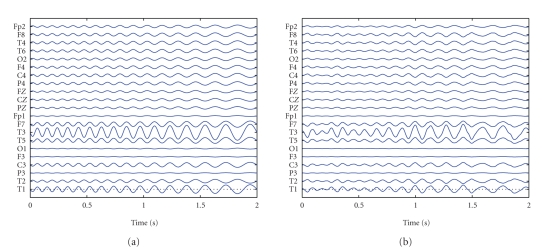
(a) The simulated frequency-modulated signal used in Figure 8 (b) The reconstructed atom.

Figure 8 shows the dipole localisation error in function of the SNR when the simulated epileptic signal changed in frequency and amplitude during the considered 2 seconds. The figure strongly resembles Figure 6(a). This means that, although the signal is not well localised in frequency, the decomposition still reliably detects the correct location. This does not mean that the seizure activity is fully separated into one atom. When we looked at the frequency component of the epileptic atom, this component had maximal values around 6 Hz, that is, the average of the start (8 Hz) and end frequency (4 Hz), while the frequency component in the first simulation peaked around 4 Hz. When the epileptic atom is reconstructed (see Figure 9), the change in frequency is not captured and the reconstruction is poor in the beginning and at the end. This is also reflected by a lower Candecomp fit percentage. In the first simulation, the fit percentage was about 75%, while in this simulation only 58% of the activity could be modeled. However, the best trilinear approximation captures a good localisation.

**Figure 10:**
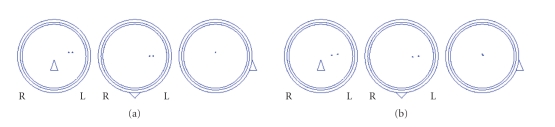
(a) The original dipole localisation of two simulated dipoles. (b) The dipole localisation when three atoms were estimated with Candecomp.

Figure 10 shows in (a) the simulated localisation of two close dipoles and in (b) the estimated localisation with the proposed method at an SNR of 0.7. The Corcondia [30] indicated that three atoms were the correct number of atoms for this simulated EEG. Two of them corresponded to the 2 dipolar foci. The localisation error was for both sources about 5 mm, which indicates that a reliable separation and localisation was obtained.

**Figure 11:**
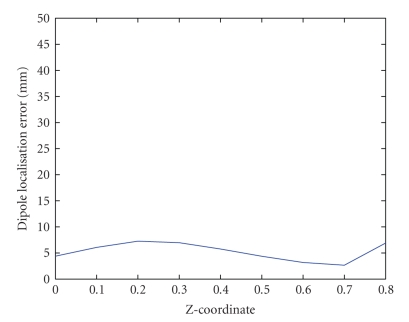
The dipole localisation error as a function of the z-coordinate of the dipole, in order to assess the influence of the depth of the dipole location.

The dipole localisation error as a function of the position of the dipole is shown in Figure 11.

**Figure 12:**
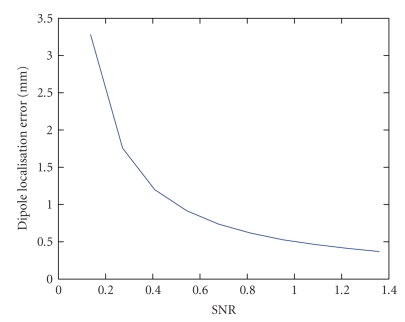
The dipole localisation error as a function of the noise level, when the EEG is recorded with 148 electrodes.

The last figure, Figure 12, shows the dipole estimation error when 148 electrodes are used to acquire the EEG. It can be seen that with a high spatial sampling, the estimation accuracy became about 1 mm.

## 4. DISCUSSION

In [24], we introduced an automatic, fast, and sensitive method for visualizing the ictal onset zone. The method was based on the multiway Candecomp of wavelet-transformed EEG in distinct “atoms.” After the decomposition, one atom could be identified as the epileptical atom, and the spatial component of this atom revealed the focus. The method was also validated on a large number of in vivo seizures, and was not influenced by the presence of strong artifacts. However, in that study, the extracted localising information was limited to the 2D potential distribution of epileptic activity over the electrodes. In the present study, we looked at the 3D localisation in a spherical head, and investigated the localising accuracy of a dipolar source fitted to the extracted potential distribution.

It is known that an infinite number of internal electrical currents correspond with exactly the same potential distribution on the scalp. The discussion if dipolar sources are superior to distributed sources is beyond the scope of this study. We chose the dipolar source because it is most popular. It is known that the generator of ictal activity can be an extended area, and that a dipole situated in a certain region should be considered as the center of mass of a larger activated brain region [7]. In [25], source densities were computed after Candecomp.

We present here the framework for seizure onset localisation with Candecomp as preprocessing step for EEG source localisation. In fact, we focussed in the paper on seizure activity. However, the method can also be used to localise all origins of oscillatory activity. We have shown that in a spherical head model with realistically simulated EEG, our algorithm correctly localised the seizure-related atom with an accuracy of about 5 mm, even at SNR ratios that are lower than one encounters during real ictal recordings. SNRs below 1 mean that the signal contains more noise than signal (see, e.g., Figure 5). Although the shape of seizure activity will not be perfectly sinusoidal, we have shown that the exact shape of the seizure signal did not really influence the localisation accuracy. In a second simulation, we have shown that the localisation error does not depend on the frequency of the epileptic signal, and that overlapping frequency content of signal and noise, representing muscle artifacts, does not lower the reliability of the decomposition. The third simulation investigated a more challenging, but maybe more realistic situation in which the frequency of the seizure changed during the considered time interval. The resulting atom could not fully capture the exact frequency-varying signal, as indicated by a lower fit-percentage of Candecomp and the reconstructed epileptic signal. However, the best trilinear approximation still reliably localised the signal. We should emphasize that Candecomp is an interesting decomposition method due to its uniqueness properties. However, the trilinear decomposition in space-time-frequency components really restricts the activity that can be fully captured. When the frequency content changes in time at a fixed position, the exact signal will not be fully separated. However, the best trilinear approximation will separate a rhythmical signal at the correct location. A similar result is observed when a moving dipole was simulated. Moving activity cannot be captured with a trilinear model, but the best approximation will result in an “average” localisation. The fourth simulation showed that the localisation error is quite insensitive to dipole localisation. In [47], it was observed that dipoles closer located to the scalp, are slightly better estimated due the higher SNR associated with higher dipoles. However, in our simulation this effect is negligible. We investigated also the situation in which two dipolar sources generating the same signal were placed near each other. This simulates multifocal epilepsy. The Corcondia [30] indicated that three atoms were the correct number of atoms for this simulated EEG. Two of them corresponded to the 2 dipolar sources. This example illustrates the interesting uniqueness property of Candecomp [30] for EEG source localisation. When matrix decomposition techniques like SVD or independent component analysis (ICA) would have been used to decompose the EEG, only 1 rhythmical source would be extracted as the 2 simulated sources are not independent nor uncorrelated. It would then not be obvious to determine the correct number of dipoles. In our approach, Candecomp determines the optimal number of components and only 1 dipole will correspond to each atom. Tensor decomposition techniques offer clearly advantages over matrix decomposition techniques as preprocessing technique for EEG source localisation. The last simulation assessed the accuracy when more electrodes are used. It is known that dipole localisation based on 21 electrode measurements gives only an approximate indication of source localisation. However, using 148 electrodes can reduce the dipole estimation error to less than 1 mm at the same low SNR's. So we think it is worth to record the EEG with denser spatial sampling.

The current simulation study is the most reliable validation of our method. In the future, we plan to validate our method on in vivo seizures with a gold standard. This gold standard can be intracranial EEG, ictal SPECT, or the site of epilepsy surgery in patients who were rendered seizure free. Comparing the estimated dipole localisation to other data, like ictal SPECT or MR-visible lesions, however, will be biased by the accuracy of the onset delineation with these diagnostic tools. We anticipate that the higher sensitivity and objectivity of our Candecomp method as compared with visual assessment of the ictal EEG's will improve and streamline the noninvasive presurgical evaluation of patients with refractory partial epilepsy.
